# Reoccurring Bovine Anthrax in Germany on the Same Pasture after 12 Years

**DOI:** 10.1128/jcm.02291-21

**Published:** 2022-03-16

**Authors:** Peter Braun, Wolfgang Beyer, Matthias Hanczaruk, Julia M. Riehm, Markus Antwerpen, Christian Otterbein, Jacqueline Oesterheld, Gregor Grass

**Affiliations:** a Department of Bacteriology and Toxinology, Bundeswehr Institute of Microbiology, Munich, Germany; b Department of Livestock Infectiology and Environmental Hygiene, Institute of Animal Science, University of Hohenheim, Stuttgart, Germany; c Bavarian Health and Food Safety Authority, Oberschleißheim, Germany; d Local Veterinarian Unit, Rosenheim, Germany; University of Iowa College of Medicine

**Keywords:** *Bacillus anthracis*, anthrax, outbreak, phylogenetics, detection assay

## Abstract

The zoonotic disease anthrax, caused by the endospore-forming bacterium Bacillus anthracis, is very rare in Germany. In the state of Bavaria, the last case occurred in July of 2009, resulting in four dead cows. In August of 2021, the disease reemerged after heavy rains, killing one gestating cow. Notably, both outbreaks affected the same pasture, suggesting a close epidemiological connection. B. anthracis could be grown from blood culture, and the presence of both virulence plasmids (pXO1 and pXO2) was confirmed by PCR. Also, recently developed diagnostic tools enabled rapid detection of B. anthracis cells and nucleic acids directly in clinical samples. The complete genome of the strain isolated from blood, designated BF-5, was DNA sequenced and phylogenetically grouped within the B.Br.CNEVA clade, which is typical for European B. anthracis strains. The genome was almost identical to BF-1, the isolate from 2009, separated only by three single nucleotide polymorphisms (SNPs) on the chromosome, one on plasmid pXO2 and three indel regions. Further, B. anthracis DNA was detected by PCR from soil samples taken from spots in the pasture where the cow had fallen. New tools based on phage receptor-binding proteins enabled the microscopic detection and isolation of B. anthracis directly from soil samples. These environmental isolates were genotyped and found to be identical to BF-5 in terms of SNPs. Therefore, it seems that the BF-5 genotype is currently the prevalent one at the affected premises. The area contaminated by the cadaver was subsequently disinfected with formaldehyde.

## INTRODUCTION

Bacillus anthracis, the causative agent of anthrax, resides dormant in soil as endospores. These spores can resurface after heavy rains (1) or, e.g., as a result of disturbances of animal burial sites (2). Typically, susceptible grazing mammals become infected by ingesting spore-contaminated soil. The anthrax pathogen is notorious for unexpectedly re-emerging after years or decades of inactivity at previous outbreak sites (1). Such events include outbreaks in Sweden (2), Siberia (3), and Italy (4, 5). In Germany, anthrax is very uncommon. The last human infections in 2012 were associated with illicit consumption of heroin allegedly contaminated with B. anthracis spores (6–8). Animal cases are equally rare, with small-scale bovine outbreaks recorded in 2009 (9), 2012 (10), and 2014 (11). While these animal cases involved B. anthracis genotypes common in Germany, the human cases raised concern, as the genotypes involved were distinct from any known German isolate but closely related to strains from the Near and Middle East (12). Likely, spores of this genotype were introduced via drug-trafficking activities involving contaminated by-products en route (6, 12). Rapid identification and genotyping of new outbreak isolates are thus important for differentiating natural, reoccurring outbreaks of domestic strains from deliberate release or accidental contamination.

Therefore, occurrence of bovine anthrax in August of 2021 raised initial alarm. However, this outbreak affected the same premises as in 2009. Back then, four heifers had succumbed to the disease and one was euthanized (13). Now, a gestating cow fell with strong suspicion of anthrax.

The genome (BF-1) of the 2009 anthrax outbreak has been published (9). This genome is closely related to other isolates of the B-branch phylogeny of B. anthracis (B.Br.CNEVA) (14). The B.Br.CNEVA genotype seems to be typical in mountainous areas in central Europe from France (14) to Slovakia (14) and from Sweden (2) to Switzerland (15). Also, to this group belongs a historical genome reconstructed from a microscopy slide prepared in Germany in 1878 featuring B. anthracis-infected dried cow blood (14).

In this report, we describe the investigation of a rare reoccurring German anthrax outbreak in southern Bavaria. Rapid detection of B. anthracis associated with anthrax outbreak events using species-specific means of identification is paramount for initiation of infection control countermeasures. Additional genomic analysis of the causative agent may help differentiate between natural infection and deliberate release of the pathogen. The aim of this study was thus the unambiguous identification of B. anthracis with a diverse set of diagnostic tools targeting the anthrax pathogen’s nucleic acids and proteins. Because of the very close spatial occurrence of the 2009/2021 outbreaks, the question of whether the B. anthracis strains involved were identical or different arose. We thus analyzed the genome sequence of the 2021 outbreak isolate and offer conclusions regarding the phylogenetic relation of this B. anthracis strain to closely related strains.

## MATERIALS AND METHODS

### Bacterial culture and inactivation.

B. anthracis strain Sterne (positive control) (16) and Bacillus cereus ATCC 10987 (negative control) were grown on Columbia blood agar (Becton Dickinson, Heidelberg, Germany) or trimethoprim-sulfamethoxazole-polymyxin blood agar (TSPBA) (17). B. anthracis was chemically inactivated with 4% (vol/vol) Terralin PAA (Schülke & Mayr GmbH, Norderstedt, Germany), as described in reference 18. Blood samples were inactivated within a class III biological safety cabinet at the Bundeswehr Institute of Microbiology biosafety level 3 (BSL-3) facility by adding 50 mL 4% (vol/vol) Terralin PAA to 0.5 mL blood. After incubation at room temperature for 30 min, samples were washed twice by centrifugation (5,000 × *g*, 5 min) with 10 mL phosphate-buffered saline (PBS) and finally resuspended in 0.5 mL PBS.

### Initial carcass samples, diagnostic PCR for B. anthracis, and microscopy.

Blood samples from the left nostril of the cow carcass were taken and transferred to the federal state veterinary laboratory and the Bundeswehr Institute of Microbiology for further analysis. Sample culture was conducted on Columbia blood agar, and the culture was grown overnight at 37°C. A single colony with typical growth morphology was cultivated, named BF-5, and used for DNA preparation (Qiagen, Hilden, Germany). PCR was performed for chromosomal markers and both virulence plasmid markers (pXO1 and pXO2) as described in the manufacturer’s instructions (RealStar anthrax PCR kit 1.0; Altona, Hamburg, Germany).

For direct PCR-based detection of B. anthracis in blood samples, 100 μL inactivated blood sample was incubated at 95°C for 10 min to lyse cells and centrifuged at 5,000 × *g* for 2 min. Aliquots of 5 μL of the supernatant were then used as templates for 16S rRNA single nucleotide polymorphism (SNP) PCR or 16S rRNA SNP reverse transcription-PCR (RT-PCR) performed as described in reference 19. Alternatively, total nucleic acid extractions of blood samples were used as templates. A MasterPure complete DNA and RNA purification kit (Lucigen, Middleton, WI, USA) was used for extraction of DNA and RNA from blood samples according to the manufacturer’s instructions for whole-blood samples.

For microscopic detection of B. anthracis from blood samples, the receptor binding protein (RBP) derivative RBP_λ03Δ1–120_ was used. A volume of 0.5 mL blood was inactivated, repeatedly washed with PBS, and mixed with 1 μg mCherry-RBP_λ03Δ1–120_ protein (18). Fluorescence microscopy was conducted as described in reference 18.

### Collection of soil samples.

On 6 September 2021, soil samples were collected from four spots corresponding to the head and tail area where the deceased cow had fallen and subsequently exuded spore-contaminated blood onto the pasture. Because of heavy rains in the area in the meantime (>50 L/m^2^), samples were collected from approximately 10 cm below the surface. Each sample comprised duplicate 50-mL conical tubes half-filled with soil (about 50 to 70 g). Samples were stored at ambient temperature.

### Soil sample analysis by PCR and culturing of B. anthracis.

Soil samples for PCR analysis were processed as described in reference 20. Briefly, three aliquots of soil samples (10 g) were resuspended in 20 mL of sterile water with glass beads (diameter, 5 mm) and mixed overnight at room temperature. Two of the aliquots were spiked beforehand with spores of strain B. anthracis Sterne 34F2 for quantification (2 × 10^2^ and 5 × 10^2^ spores per sample). The suspensions were filtered through sterile gauze to remove soil particles and other rough materials. After centrifugation at 4,000 × *g* for 15 min, the pellet was washed three times in sterile water and finally resuspended in 5 mL distilled water. This suspension was heated to 65 to 70°C for 30 min to inactivate vegetative cells. Volumes of 250 μL each were plated onto four semiselective agar plates (TSPBA) (21). Plates were incubated overnight at 37°C. Then, the bacterial lawn from each plate was scraped off and resuspended in 4 mL of 0.9% (wt/vol) NaCl solution. An aliquot (ca. 1 mL) of this suspension was boiled for 20 min in a heating block to release DNA from cells and centrifuged at 12,000 × *g* for 15 min, and the supernatant was filtered through a 0.45-μm luer lock filter. Aliquots of 5 μL of the filtered supernatant were used for PCR analysis (20). If they were PCR positive, dilutions of the original suspension were plated and grown on TSPBA (17) for isolation and verification of suspected B. anthracis colonies (20). DNA from a picked colony was tested by PCR for B. anthracis-specific markers as described in reference 1. Additional enrichment of B. anthracis from soil samples was achieved by culturing on semiselective CEFOMA (Bacillus cereus sensu lato group-specific antibiotics, fosfomycin, macrolides agar) as described in reference 22.

### Enrichment of B. anthracis from soil samples by magnetic separation and culturing.

For enriching B. anthracis from possibly spore-contaminated soil samples, a newly developed magnetic bead-assisted magnetic separation method was applied. In this approach, RBP_λ03Δ1–120_ (18) was repurposed to capture B. anthracis from soil. In short, Strep-Tactin XT protein (IBA GmbH, Göttingen, Germany) was coupled to magnetic beads (Dynabeads M-280, tosyl activated; Thermo Fisher, Dreieich, Germany). Then, RBP_λ03Δ1–120_ protein was attached to this Strep-Tactin XT via the Twin Strep-tag epitope. Soil was processed as described in reference 17; i.e., a soil sample was shaken in PBS buffer with 0.5% (vol/vol) Tween 20 to solubilize spores. The sample was gently centrifuged to remove solid material, and the crudely cleared supernatant was incubated at 62°C for 20 min to inactivate vegetative cells. The supernatant was mixed 1:10 with brain heart infusion broth (Merck, Darmstadt, Germany) with 10% (vol/vol) fetal calf serum (Merck) and incubated to allow spores to germinate and develop into vegetative cells. This germination culture was mixed and incubated with the RBP-loaded magnetic beads to separate B. anthracis spores from the liquid. Separation was accomplished using a magnetic stand (Thermo Fisher). Beads were washed and finally plated onto TSPBA or Columbia blood agar plates (Becton Dickinson). Colonies were evaluated after overnight incubation at 37°C. Full details on the method will be published elsewhere.

### Rapid prescreening of candidate B. anthracis colonies.

Blood samples from the carcass or colonies suspicious for B. anthracis obtained after enrichment from soil samples were subjected to a colorimetric enzyme-linked phage receptor binding protein assay (ELPRA) as described in reference 23. In short, the one-step assay version was applied that utilizes recombinant horseradish peroxidase (HRP)-coupled RBP_λ03Δ1–120_. Candidate colony material or blood was inactivated, washed twice with PBS, and incubated with 0.1 μg of HRP-RBP_λ03Δ1–120_. Samples were repeatedly washed with PBS, and the pellet was resuspended in 50 μL SeramunBlau slow (containing 3,3′,5,5′-tetramethylbenzidine) peroxidase substrate (Seramun Diagnostica, Heidesee, Germany). Blue color development was monitored for several minutes and photodocumented. Inactivated sheep blood served as a negative control.

### High-quality DNA preparation from B. anthracis colony material and confirmative PCR.

Single bacterial colonies grown on semiselective agar (TSPBA) were chemically inactivated with 4% Terralin PAA, and DNA was isolated using the MasterPure DNA purification kit for Gram-positive organisms (Lucigen, Middleton, WI, USA) with minor modifications as described in reference 24. DNA concentrations were quantified using the Qubit double-strand DNA (dsDNA) HS assay kit (Thermo Fisher Scientific, Darmstadt, Germany), according to the supplier’s protocol. For confirmation of B. anthracis DNA via PCR, the chromosomal marker *dhp61* was used as described previously (25). DNA preparations were stored at −20°C until further use.

### Whole-genome sequencing.

Nanopore sequencing was performed using SQK-LSK109 chemistry on a R10.3 SpotON flow cell on the GridION system (Oxford Nanopore Technologies, Oxford, UK) running system software MinKNOW 21.05.8. A total of 350,000 reads were generated using the implemented “superaccurate base calling” model. For increasing the assembly efficacy, the number of reads was down-sampled to 104,110 reads (*N*_50_ of 10.01 kb; mean raw quality score of Q13.5). After processing using Flye assembler V2.9 (26), three circularized high-quality replicons, corresponding to the chromosome (5,213,322 bp; coverage, 174-fold) as well as both plasmids pXO1 (181,920 bp; coverage, 614-fold) and pXO2 (94,735 bp; coverage, 491-fold), were obtained. The scaffolds were manually checked for contaminant reads and annotated automatically by the NCBI Prokaryotic Genome Annotation Pipeline (27) after submission. CanSNPer (v1.0.10) (28) was used to classify and subsequently assign the corresponding canonical SNP (canSNP) group B.Br.CNEVA to this genome.

### Analysis of whole-genome sequencing data and SNP-calling.

For rapid core chromosome multiple-alignment, the Parsnp tool from the Harvest Suite (version 1.1.2) was used (29). For this, a chromosome data set, representing genomes from public databases (Table S1) and the newly sequenced strains of B. anthracis, were aligned against the chromosome of B. anthracis Ames ancestor (NC_007530) as a phylogenetic outgroup using Parsnp (parameters -c -e -u -C 1000). To export the identified SNP positions, HarvestTools (version 1.2) from the same software suite was used to create a vcf (variant calling file) listing all SNP positions. In order to enhance data quality, chromosome regions with closely adjacent SNPs (<10 bp distance) and positions harboring undefined nucleotides (“N”) were removed. This curated vcf was used as input for HarvestTools to compile a multi-FASTA file from the chromosome data set, comprising the concatenated SNPs as a multiple-sequence alignment. This concatenated sequence information was used to calculate a maximum-likelihood tree in MEGA X (version 10.0.5) (30, 31). A minimum spanning tree was computed in BioNumerics 6.6 (Applied Maths, Sint-Martens-Latem, Belgium) from the SNP vcf (in binary format) as input and manually edited (using PowerPoint 2016, Microsoft) for style.

### Analysis of the distribution of SNPs specific for B. anthracis strain BF-5 in other isolates.

DNA of several additional clones retrieved from soil sampling was subjected to SNP analysis. For this, regions covering the SNP regions identified by genome sequencing were PCR amplified (primers are listed in Table S2) and Sanger DNA sequenced (Sequencing was performed by Eurofin Genomics, Ebersbach, Germany). DNA sequence analysis was conducted with Geneious Prime (Biomatters, USA).

### Data availability.

All data generated or analyzed during this study are included in this published article, and its supplemental information files are publicly available in the NCBI Sequence Read Archive (SRA) repository (BioProject PRJNA171093).

## RESULTS

### B. anthracis infection in a deceased cow was confirmed by initial *in situ* and PCR diagnostics.

**FIG 1:**
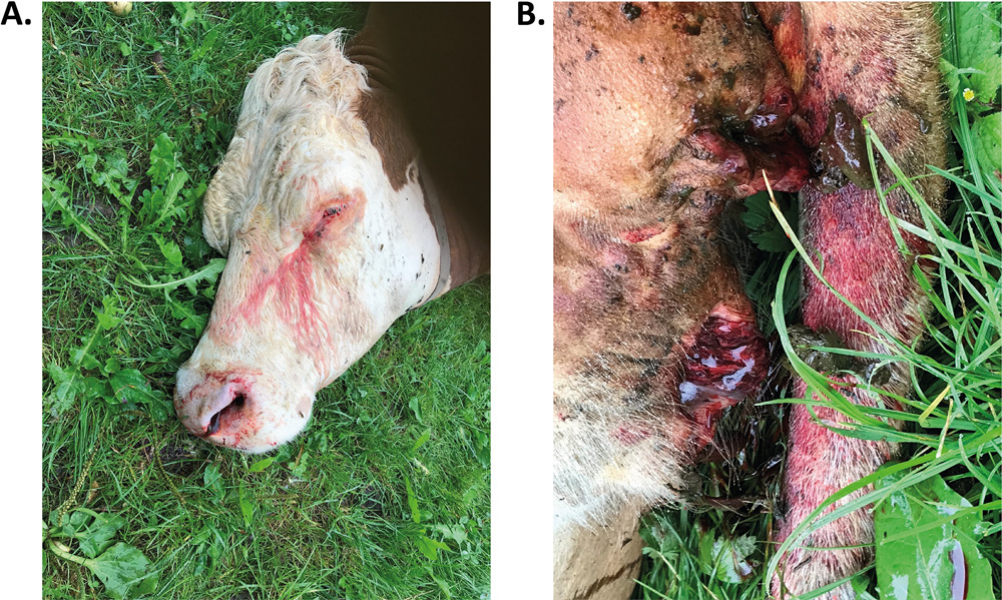
*In situ* presentation of a cow that died of anthrax. A 2-year-old gestating cow succumbed to anthrax on a pasture in southern Bavaria (Germany) in August of 2021 (A and B). Close-up of the head with bloody discharge from the eyes and left nostril (A) and rear view with bloody anus and vagina (B).

Veterinary examination of a deceased gestating cow on a pasture near Rosenheim (Bavaria, Germany) on 24 August 2021 raised suspicion of anthrax infection due to the disease-typical symptoms, i.e., sudden death and bloody discharge from all body orifices, including nostrils, eyes, vagina, and anus (Fig. 1A and B). PCR of DNA isolated from colonies with typical morphology grown after cultivating blood from the deceased animal gave positive results for diagnostic B. anthracis markers, the *dhp61*, *pag*, and *cap* genes (data not shown). Thus, anthrax disease was confirmed and an official diagnostic report released.

### Detection of B. anthracis directly in blood samples by phage RBP-based reporter and 16S rRNA SNP (RT-)PCR.

**FIG 2:**
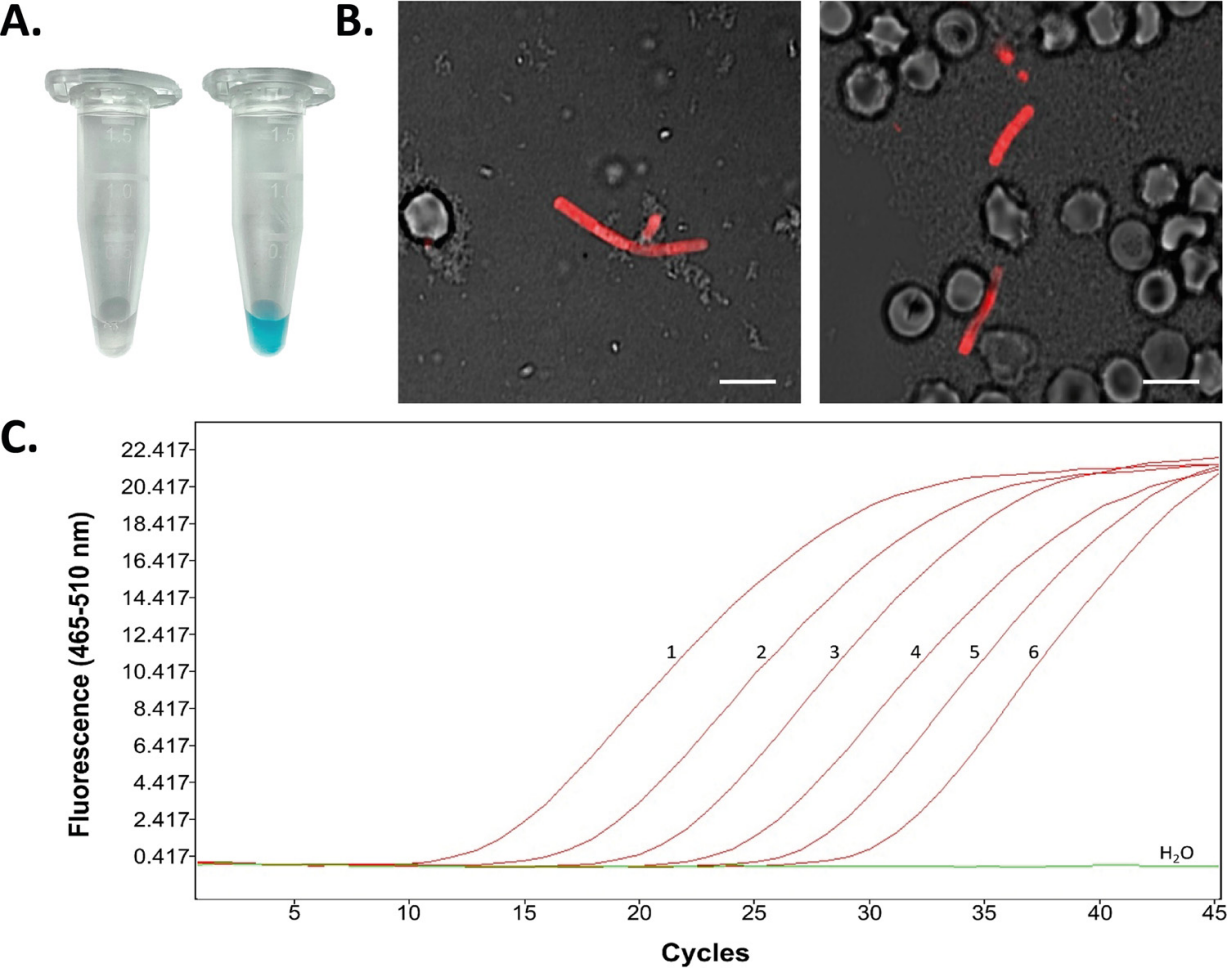
Direct detection of B. anthracis cells in blood from a diseased cow and molecular PCR diagnostics. (A) HRP-conjugated RBP_λ03Δ1–120_ was added directly to inactivated blood (taken from the carcass’ left nostril) (right tube) as well as to inactivated sheep blood, which served as a negative control (left tube). After washing, chromogenic HRP substrate was added, and color development was photodocumented after 1 min. (B) Recombinant fusion protein mCherry-RBP_λ03Δ1–120_ was added to 100 μL of blood and directly subjected to fluorescence microscopy. Shown are merged images of transmission and fluorescent light (wavelengths: excitation, 594 nm; emission, 610 nm). Bar, 5 μm. (C) Dilutions of the inactivated cow blood (curve 1, 1:10; curve 2, 1:100; curve 3, 1:1,000) as well as dilutions of isolated DNA (curve 4, 1:10; curve 5, 1:100; curve 6, 1:1,000) from the blood samples were subjected to 16S rRNA SNP-PCR. Shown are representative real-time PCR amplification curves.

Independent of initial diagnostic PCR analysis performed by state health authorities, blood taken from the left nostril of the carcass (Fig. 1A) was inactivated and subjected to recently developed ultrasensitive 16S rRNA SNP (RT-)PCR (19) and phage RBP reporter-based rapid detection assays (18). Results confirmed the previous PCR tests, as phage RBP_λ03Δ1–120_ reporter-based ELPRA gave positive results when inactivated blood samples from the carcass were tested (Fig. 2A). Using fluorescence microscopy, the mCherry-RBP_λ03Δ1–120_ reporter was found to specifically bind to bacterial chains in blood samples, as evidenced by red fluorescence (Fig. 2B). This indicated that the detected cells were indeed very likely B. anthracis. Of note, these phage RBP-based tests can be performed in just a few minutes. Using 16S rRNA SNP-PCR, specific detection of B. anthracis nucleic acids directly in the blood samples derived from the carcass, as well as from nucleic acid extractions thereof, was also accomplished (Fig. 2C). Dilutions (1:10 to 1:1,000) of the inactivated blood sample (without prior nucleic acid extraction) yielded cycle threshold (*C_T_*) values from 24.9 to 31.7. Conversely, dilutions of total nucleic acid extracted from the same blood sample yielded *C_T_* values from 13.9 to 21.5 in tests for DNA only (Table S3). When these total nucleic acid preparations (containing DNA and RNA) were subjected to 16S rRNA SNP RT-PCR, the same samples (dilutions 1:10 to 1:1,000) yielded even lower *C_T_* values (9.7 to 17.8) (Table S3). This is because the ultrasensitive RT version of the PCR detects not only 16S rRNA genes of B. anthracis but also their transcripts, which are more abundant in growing cells than their respective gene copies.

### B. anthracis strains BF-1 and BF-5 are clonal, very closely related outbreak strains.

Genomic DNA of B. anthracis strains BF-5 was subjected to sequencing resulting in three contigs (chromosome, plasmid pXO1 and pXO2) (accession numbers CP089993 to CP089995). Comparison of the genomes of B. anthracis strains BF-1 and BF-5 revealed that both strains were exceptionally similar (Table 1). The chromosome of BF-5 featured only three SNPs and two single nucleotide repeat (SNR) differences (both SNRs in noncoding regions with deletions of a single T). While plasmid pXO1 was identical, pXO2 harbored a single additional SNP and SNR insertion (T) in three identical repeat regions, respectively. This clonality of the two outbreak strains clearly supported the hypothesis that a hitherto-nonlocalized source of unknown origin of contamination exists on site. This source is very likely the cause of repeated infection of grazing cows on this pasture.

**TABLE 1:** DNA sequence differences between genomes of B. anthracis BF-1 and BF-5

Reference (BF-1)	Position	BF-1 nucleotide sequence (ancestor state)	BF-5 nucleotide sequence (derived state)	Kind of change
CP047131.1 (chromosome)	519877	C	T	SNP (SNP1)
	1434950	CTTTTTTTTTTTTTTGTAAATAA	CTTTTTTTTTTTTTGTAAATAA	Deletion
	1625072	A	C	SNP (SNP2)
	1878269	GTTTTTTTTTTTTTTTGTAAAATTAA	GTTTTTTTTTTTTTTGTAAAATTAA	Deletion
	2472315	T	C	SNP (SNP3)
CP047133.1 (plasmid pX02)	29759, 31759, 30759	CTTTTTTTAT	CTTTTTTTTAT	Insertion
	62640	A	G	SNP (SNP4)

### Phylogenetically, strains B. anthracis BF-1 and BF-5 group with strains from the Austrian state of Tyrol.

**FIG 3:**
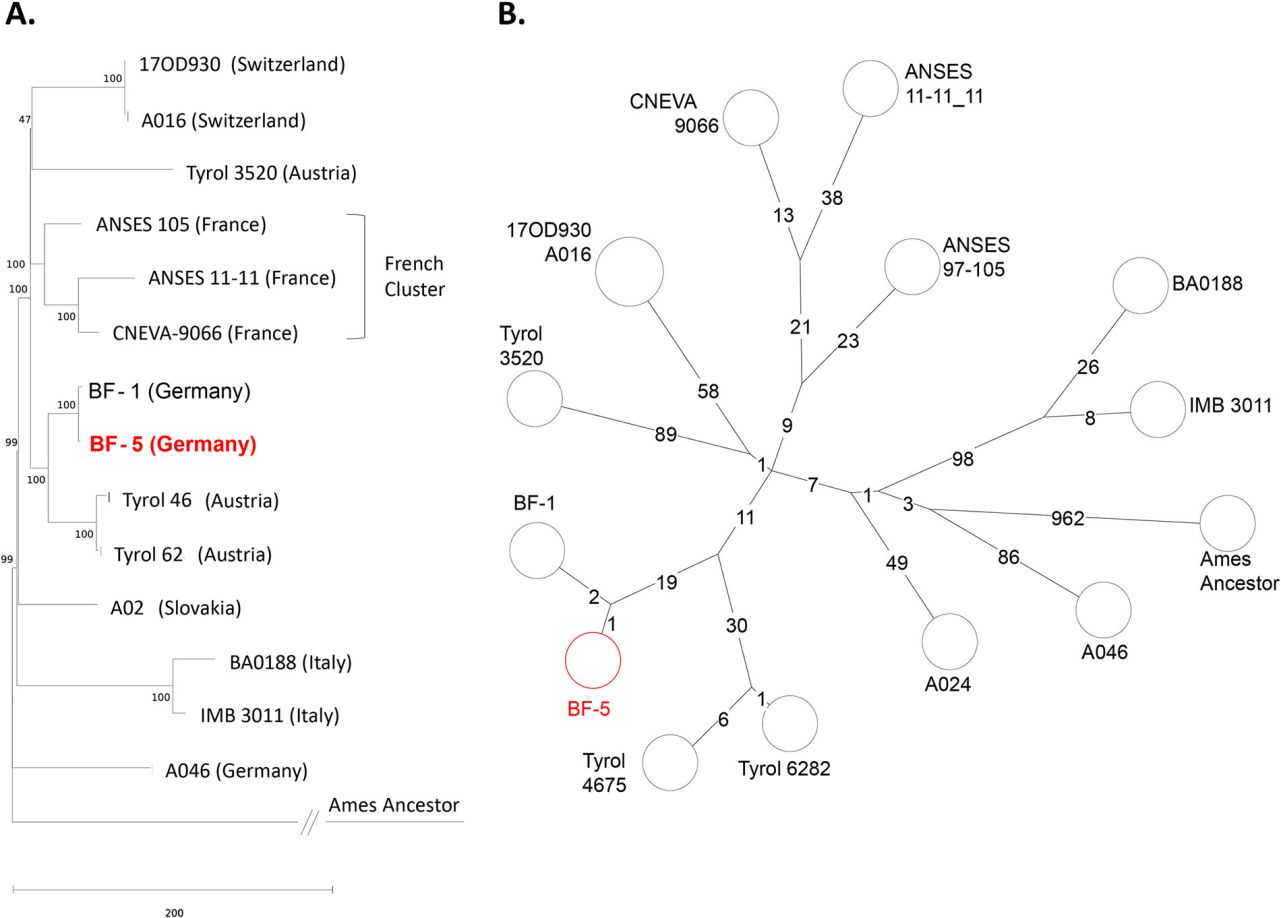
Phylogeny of new B. anthracis isolate BF-5 among its close relatives of the B.Br.CNEVA canonical SNP (canSNP) clade. (A) Rooted phylogenetic tree of representatives of the B.Br.CNEVA canSNP clade of B. anthracis. The tree is based on 1,558 chromosomal SNPs used to construct a maximum-likelihood tree (bootstrap confidence from 500 permutations was generated, and the tree with the highest likelihood is shown). Isolate names and countries of origin are at branch termini (red, sequenced in this study; black, sequences from public databases) (Table S1). (B) Minimum‐spanning tree of close relatives of strain BF-5 within the B.Br.CNEVA canSNP clade of B. anthracis derived from chromosomal SNPs. Indicated are numerical SNP differences (logarithmic scale) between chromosomes. Both trees are rooted to the reference chromosome, B. anthracis strain Ames ancestor, which belongs to the A.Br.Ames canSNP clade.

The canSNP type of B. anthracis BF-5 was determined, assigning the strain to the B.Br.CNEVA clade (32). Chromosomal sequence analysis inferred the phylogenetic placement of strain BF-5 in a cluster of central European B. anthracis strains within the B.Br.CNEVA clade. As expected from Table 1, the closest relative was strain BF-1 (Fig. 3). Other close relatives were Tyrol 4675 and Tyrol 6282, isolated in the Austrian state of Tyrol from 1988 and 1979, respectively. Strains from a large French B.Br.CNEVA cluster (only three representatives are shown in Fig. 3) as well as strains from Switzerland, Slovakia, Germany, and Italy were more distantly related. Not shown are additional B.Br.CNEVA genomes phylogenetically more distantly related to the focus strain, BF-5. Notably, there is a polytomy at the base of the French cluster, the clade comprising strains A016/17OD930 and Tyrol 3520 and the clade featuring BF-1 and BF-5 as well as Tyrol 4674 and Tyrol 6282 (Fig. 3). This clearly suggests a common ancestor of all the strains.

### Both classical, established methods and novel phage RBP reporter fusions enable direct detection and isolation of B. anthracis from soil samples.

**FIG 4:**
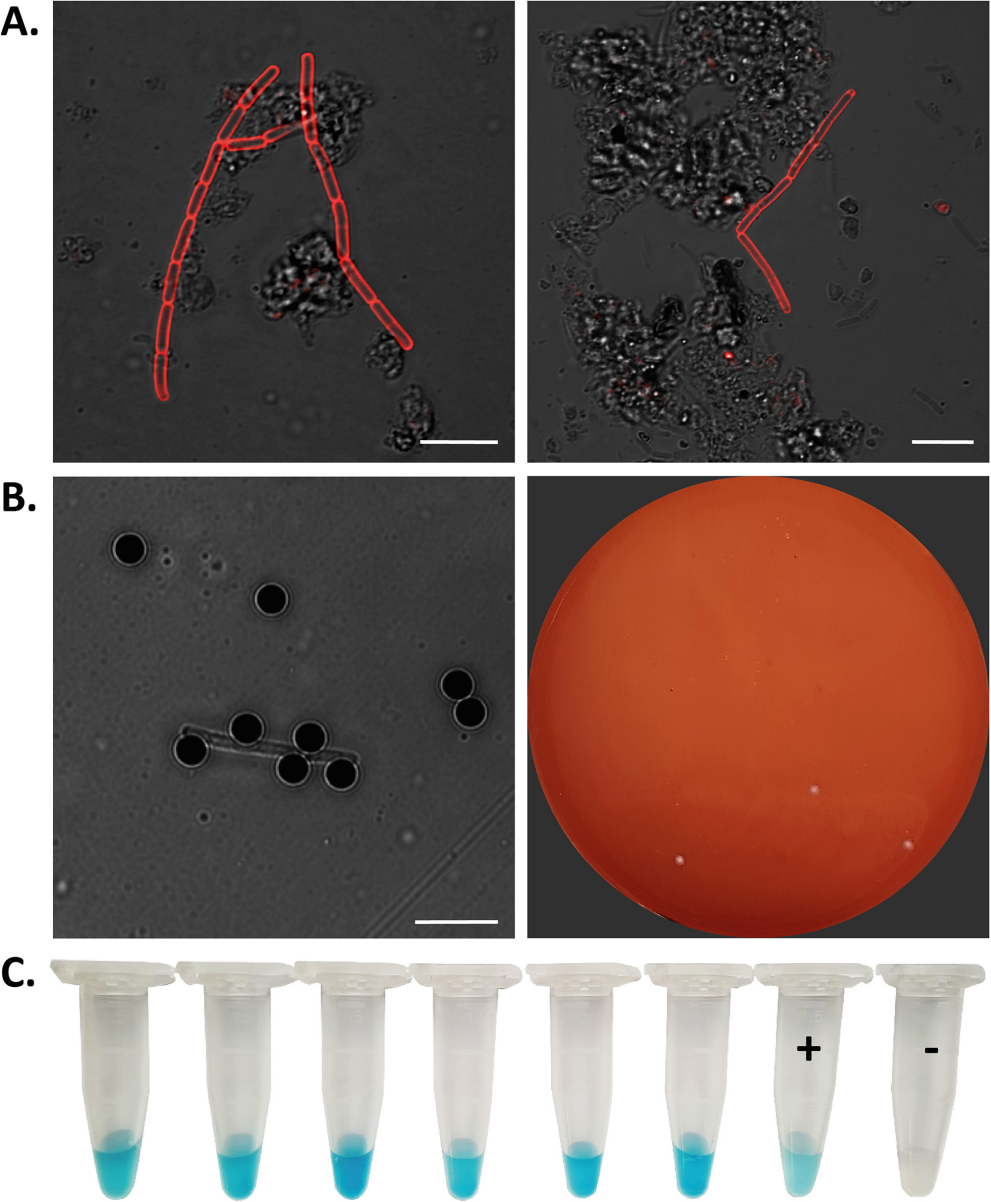
Direct detection and isolation of B. anthracis from contaminated soil samples associated with a deceased cow. Soil samples were shaken in PBS-Tween (PBST) buffer to solubilize spores, centrifuged and the supernatant mixed with brain heart infusion (BHI) broth containing fetal calf serum and incubated to allow spores to germinate. (A) Recombinant fusion protein mCherry-RBP_λ03Δ1–120_ was added to preincubated soil supernatants and directly subjected to fluorescence microscopy. Shown are two merged images of transmission and fluorescent light (wavelengths: excitation, 594 nm; emission, 610 nm). Bar, 5 μm. (B) Magnetic beads coupled with RBP_λ03Δ1–120_ were added to preincubated soil supernatants to capture B. anthracis cells. A sample was taken for bright-field microscopy (left) (bar, 5 μm), and the remainder of the bead suspension buffer-washed washed, plated on blood agar plates, and incubated at 37°C overnight (right). (C) Rapid RBP reporter-based assay on inactivated suspicious colony material from enrichment plates. Inactivated colony material was incubated with RBP_λ03Δ1–120_ covalently linked to horseradish peroxidase for colorimetric identification with chromogenic substrate. The positive control (+) was B. anthracis Sterne, and the negative control (−) was B. cereus ATCC 10987. Results were scored after about 1 min as positive (blue color development) or negative (no color development).

Soil samples were retrieved (single samples each) from the site of the carcass from depths of about 5 to 10 cm. This corresponded to soil positions close to those of head and anus of the deceased cow (Fig. 1A and B). The established analysis methods yielded positive PCR results after cultivation of original soil materials. Isolated colonies with typical morphology of B. anthracis were positive in PCR for *pagA*, *capC*, and *saspB* (data not shown). The novel, phage protein-based magnetic enrichment approach performed equally well but can be completed in a much shorter time: To screen the possibly contaminated soil samples for B. anthracis spores, mCherry-RBP_λ03Δ1–120_ was added to soil supernatants preincubated with germination medium, and the samples were subjected to fluorescence microscopy. With this method, cells of B. anthracis could be detected directly in soil samples as cell chains emitted strong red fluorescence derived from the attached RBP reporter (Fig. 4A). While the presence of B. anthracis was indicated by fluorescence microscopy, isolation of B. anthracis from soil samples was achieved using magnetic beads coupled with RBP_λ03Δ1–120_. After binding of the cells to the RBP-loaded magnetic beads, the buffer-washed cell-bead complexes (Fig. 4B, left) were agar plated and cultured. A representative result is shown in Fig. 4B (right). While hemolytic, non-B. anthracis colonies (negative in *dhp61* PCR) occasionally also grew on the plates, suspected B. anthracis colonies showing no hemolysis were chemically inactivated and confirmed by ELPRA (Fig. 4C). Genomic DNA from six of these additional isolates was prepared for further analysis.

### Four SNPs found between B. anthracis strains BF-1 and BF-5 were interrogated in additional isolates derived from contaminated soil.

In order to determine the distribution and relative abundance of the four SNPs separating B. anthracis strains BF-1 and BF-5 (Table 1; Table S2), PCRs of the identified four SNP-regions were conducted on DNA from six B. anthracis soil isolates, and the PCR amplicons were Sanger sequenced. We did not identify any SNP differences in these six soil isolates relative to BF-5 (data not shown). Thus, these results indicate that the BF-5 genotype is the prevalent genotype at the affected pasture in 2021.

## DISCUSSION

Regarding risk assessment, reoccurrence of an anthrax outbreak after 12 years (9) at the same pasture diminished the suspicion of intentional release of the pathogen as the underlying cause. Conversely, the outbreak strongly indicated that an old anthrax focus was still active. This is reminiscent to similar situations in other regions of Europe. For instance, in Sweden, an outbreak in cattle occurred in a nature reserve in 2011. Notably, records positioned an old anthrax burial site (mid-1940s) in that area (2, 33). Remarkably, only 2 years later, an additional cow died close to this area, which had seen cattle vaccination after the 2011 outbreak (34). The complete elimination of B. anthracis spores from soil within a natural focus cannot be ensured by any decontamination measure (35). Therefore, German law considers the temporary closure of respective areas for grazing to prevent reinfection, in addition to decontamination trials (German Federal Ministry of Justice/Bundesministerium für Justiz: Verordnung zum Schutz gegen den Milzbrand und den Rauschbrand [https://www.gesetze-im-internet.de/milzbrbv/BJNR011720991.html; accessed 6 January 2022]) (35). Similar to the case at hand, genome sequencing of the two Swedish outbreak isolates from 2011 and 2013 indicated that these were clonal (2). The authors offered as plausible explanation for this genomic identity among spatially and temporally separated outbreaks: the spreading of spores by birds or wildlife. Though these Swedish outbreaks caused public alarm regarding the risk of environmental contamination (2), no more cases were reported in that region since (as of November 2021). More active is the re-emerging situation in Italy, where anthrax resurfaces repeatedly in the southern region of Basilicata (36, 37) and soils at outbreak sites have remained contaminated with viable spores for many years (4, 5). Finally, the phylogeny of B.Br.CNEVA is well characterized in France, where this lineage is dominant and ecologically established in the Alps, Pyrenees, and Massif Central (plus Saône-et-Loire) (38). In contrast to France, where all B.Br.CNEVA strains are monophyletic (38) (Fig. 3), the situation differs in Germany and Austria. Isolates from these countries are distributed across several closely related lineages branching off a very shallow polytomy (Fig. 3). This not only suggests that the B.Br.CNEVA clade was introduced by a single event into France as proposed earlier (38) but also hints at a similar process of limited introduction of the branch of B. anthracis into central Europe. In this model, an early introduction event of the pathogen occurred in Italy, Slovakia, and parts of Germany, from which, again, a likely single introduction event is linked to the ancestor of B.Br.CNEVA in France, Austria, Switzerland, and Bavaria (Fig. 3).

The genomes of strains BF-1 and BF-5 differ by only three chromosomal SNPs (Table 1). A recent genomic study on an anthrax outbreak in Italy found strains differing by up to five SNPs (39). Genome analysis for epidemiological investigation of strains associated with injectional anthrax led the authors to the conclusion that genetic variation is possibly generated as a result of infection of a single host. Nonetheless, some phylogenetic patterns might be best explained by diversity introduced through several infection cycles of B. anthracis in several hosts (8). The 2021 outbreak in Bavaria seems to follow this pattern, with only very few SNPs between strains from the same outbreak site separated by 12 years. Notably, all six isolates retrieved from soil surrounding the carcass site featured the same unique SNP positions as isolate BF-5, directly grown from the dead cow’s blood. In contrast, it is very unlikely that isolate BF-1 is a direct ancestor of BF-5. Chromosomal SNP 1 differs from the ancestor state (Ames ancestor) only in BF-5 but not in BF-1. In contrast, however, chromosomal SNP 2 and SNP 3 showed an evolved state (relative to Ames ancestor) in BF-1, while being ancestral in BF-5 (Table 1).

In order to acutely diminish the local risk of near-surface spore contamination on site, the affected pasture site where the animal fell (Fig. 1A and B) was disinfected with 10 L/m^2^ 10% (vol/vol) formaldehyde, as advised in reference 1. Obviously, this measure will be able neither to disinfect deeper soil horizons nor to eliminate the unidentified original contamination site, presumably located somewhere on the premises. Longer-term monitoring of near-surface soil on site may be able to alert authorities in case B. anthracis spores can again be detected after favorable weather conditions, e.g., heavy rains followed by mild temperatures (40). Further developments related to sensitive detection of B. anthracis in soil could facilitate the identification and elimination of the original source of spore contamination at the affected premises.

In any case, this rare outbreak provided an ideal opportunity for real-life testing of assays developed beforehand for detection and identification of B. anthracis. Direct microscopy of B. anthracis-infected blood (Fig. 2A) or germinated cells in B. anthracis spore-contaminated soil (Fig. 4A) and rapid testing of inactivated blood (Fig. 4B) and suspected colonies (23) yielded similar results with these authentic materials to those obtained with previously tested spiked-in materials (our unpublished data).
