# Supplementary material for: Reoccurring Bovine Anthrax in Germany on the Same Pasture after 12 Years
Source: J Clin Microbiol. 2022 Mar 16;60(3):e02291-21. doi: 10.1128/jcm.02291-21 (PMC8925895; doi:10.1128/jcm.02291-21)
Supplement: Supplemental File 1 — Tables S1 to S3. Download jcm.02291-21-s0001.pdf, PDF file, 0.7 MB [file jcm.02291-21-s0001.pdf]

Supplementary Material for

Reoccurring bovine anthrax in Germany on the same pasture after 12 years

Peter Braun[1], Wolfgang Beyer[2], Matthias Hanczaruk[3], Julia M. Riehm[3], Markus Antwerpen[1], Christian Otterbein[4], Jacqueline Osterheld[1] and Gregor Grass[1]*

[1]Bundeswehr Institute of Microbiology (IMB), Department of Bacteriology and Toxinology, Munich, Germany;
[2]Department of Livestock Infectiology and Environmental Hygiene, Institute of Animal Science, University of Hohenheim, Stuttgart, Germany
[3]Bavarian Health and Food Safety Authority, Oberschleißheim, Germany
[4]Local Veterinarian Unit, District Rosenheim, Germany
*Correspondence: gregorgrass@bundeswehr.org; Tel.: +49-992692-3981

Keywords: *Bacillus anthracis*, anthrax, outbreak, phylogenetic, detection assay

Running title: Recurring bovine anthrax after 12 years

Supplementary Table S1: Genome sequences accession numbers of newly sequenced and additional *B. anthracis* strains from publically available databases.

Strain designation	Country	Year	SNP-group	Accession number (chromosome)
Ames Ancestor	U.S.A.	1981	A.Br.Ames	NC_007530
**BF-5**	**Germany**	**2021**	**B.Br.CNEVA**	**SRR16572036**
BF-1	Germany	2009	B.Br.CNEVA	CP047131
Tyrol 6282	Austria	1979	B.Br.CNEVA	SRR10743038
Tyrol 3520	Austria	1980	B.Br.CNEVA	SRR10743039
Tyrol 3520	Austria	1980	B.Br.CNEVA	SRR10743039
Tyrol 4675	Austria	1988	B.Br.CNEVA	CP018903
A024	Slovakia	1972	B.Br.CNEVA	QAEL00000000
A016	Switzerland	*	B.Br.CNEVA	QAEM00000000
A046	Germany	*	B.Br.CNEVA	QAEN00000000
BA0188	Italy	2007	B.Br.CNEVA	SRR12435826
**IMB 3011**	**Italy**	**2005**	**B.Br.CNEVA**	**SRR16573065**
17OD930	Switzerland	2017	B.Br.CNEVA	SRR7100210
CNEVA-9066	France	1992	B.Br.CNEVA	NZ_AAEN01000000
ANSES 97-105	France	1997	B.Br.CNEVA	ERR1596585
ANSES 11-11_11	France	2011	B.Br.CNEVA	ERR1596595

bold: sequenced in this study; *information missing (Sahl et al., 2016**).

**Sahl JW, Pearson T, Okinaka R, Schupp JM, Gillece JD, Heaton H, et al. (2016) A *Bacillus anthracis* genome sequence from the Sverdlovsk 1979 autopsy specimens. MBio 7.

Supplementary Table S2: Primers used for SNP typing of additional isolates of *B. anthracis* BF-5 retrieved from contaminated soil at carcass site.

Primer Name (position)	Forward (5'-3')	Reverse (5'-3')
SNP 1	GCCACACTGGGACTGAGAC	GCCACCTACGTATTACCGCG
SNP 2	GAGGAAAGAGAGATGTTATTGTAGAAG	TACTCCTGGAACTCCTGAAGTAAC
SNP 3	TTGAGCCATTGTAAAAATCATCCCTTC	GTTTCTGTATCATAATATAAATAGTTGGGATG
SNP 4	ATTTCTTCTTTCAGGTACATAATAAAAGCAG	CTAAAGGAGTTGACGTATTAATGGAAG

Supplementary Table S3: Ct values of 16S rRNA SNP (RT)-PCR of total nucleic acids from blood samples.

Sample	Ct value 16S rRNA SNP-PCR	Ct value 16S rRNA SNP RT-PCR
Inactivated blood 1:10	24.9	not done
Inactivated blood 1:100	28.1	not done
Inactivated blood 1:1000	31.7	not done
Nucleic acid extraction from blood 1:10	13.9	9.7
Nucleic acid extraction from blood 1:100	18.1	12.8
Nucleic acid extraction from blood 1:1000	21.5	17.8
H_2O (negative control)	-	-

* total nucleic acids were extracted from inactivated cow blood samples.
